# Shape Sensing of Hyper-Redundant Robots Using an AHRS IMU Sensor Network

**DOI:** 10.3390/s22010373

**Published:** 2022-01-04

**Authors:** Ciprian Lapusan, Olimpiu Hancu, Ciprian Rad

**Affiliations:** Department of Mechatronics and Machine Dynamics, Technical University of Cluj-Napoca, 400114 Cluj-Napoca, Romania; ciprian.lapusan@mdm.utcluj.ro (C.L.); ciprian.rad@mdm.utcluj.ro (C.R.)

**Keywords:** sensor network, IMU sensor, robot shape sensing

## Abstract

The paper proposes a novel approach for shape sensing of hyper-redundant robots based on an AHRS IMU sensor network embedded into the structure of the robot. The proposed approach uses the data from the sensor network to directly calculate the kinematic parameters of the robot in modules operational space reducing thus the computational time and facilitating implementation of advanced real-time feedback system for shape sensing. In the paper the method is applied for shape sensing and pose estimation of an articulated joint-based hyper-redundant robot with identical 2-DoF modules serially connected. Using a testing method based on HIL techniques the authors validate the computed kinematic model and the computed shape of the robot prototype. A second testing method is used to validate the end effector pose using an external sensory system. The experimental results obtained demonstrate the feasibility of using this type of sensor network and the effectiveness of the proposed shape sensing approach for hyper-redundant robots.

## 1. Introduction

Hyper redundant robots are characterized by the ability to execute complex movements in workspaces with obstacles due to the large number of Degrees of Freedom (DoF). Usually, the number of DoF that characterized such a robot is >>6, typically above 12-DoF. As a result, the robot has the possibility to reach a certain position in the workspace in an almost infinite number of configurations for the joints values [1].

These advantages make this type of robots very appealing in certain fields where a high degree of flexibility and adaptability is required during the operations. The development of hyper-redundant robots can be traced back to 1972 when Hirose et al. built a snake-like robot prototype with 20-DoF called ACM III [2]. That prototype became the reference for this type of robots and many researchers tried over the years to improve the original concept in different ways [3].

Structurally, snake-like hyper-redundant robots (serpentine robots) can be categorized as (1) continuum/soft manipulators with an “infinite number of DoF” with a single continuous flexible backbone and (2) articulated joint-based rigid manipulators with a limited number of DoF composed of modules serially connected in hyper-redundant structures that can produce smooth curves [4,5].

In the last years, these robots were increasingly used as advanced dexterous manipulators for various tasks in different domains. For example, OC Robotics has built Series II—X125 snake-arm industrial robot that has 12 links serially connected each with 2-DoF for a total of 24-DoF. It has a total bending capability of 225 deg with a 27.5 deg per link and can manipulate payloads up to 10 kg. The robot can be used for cutting, welding, grasping, or fastening operations [6]. More recently, Martín-Barrio et al. [7] designed a discrete joint-based hyper-redundant cable-actuated robot with 7 modules serially connected for a total of 14-DoF to be used for inspection tasks in constrained environments. Shape sensing, kinematic control and remote operation in an immersive reality were the main challenges reported by the authors. Precision/vertical farming is another field with unstructured environment where hyper-redundant robots are increasingly used for soft grasping, harvesting, precision planting, precision spraying, or precision fertilizing tasks [8,9,10]. However, design, control and shape sensing are the main challenges when it comes to implement these types of manipulators in this area [11].

Hyper-redundant robots have great potential to be used in unstructured environments where highly dexterous manipulations are needed and although these robots have been used in many applications in the last decade, precise modelling, kinematic control, and mechanical design are still challenging problems as highlighted in [12,13,14,15].

A practical solution in this matter is to use an advanced real-time feedback system for shape sensing. That system should include advanced shape sensing sensors, model-based shape reconstruction techniques (kinematic models such as curve parametrization and Piecewise Constant Curvature and/or dynamic models such as Cosserat Rod, spring-mass model, and real-time finite element method [1]), robust communication protocols and data fusion algorithms to name a few. In this context, this paper addresses the issues of shape sensing for joint-based snake-like hyper-redundant robots.

As highlighted in [16,17], for shape sensing there are two types of sensors: non-contact-based (NCSSs) and contact-based shape sensors (CSSs). NCSSs include video cameras with imaging techniques such as Fluoroscopy, RaDAR and LiDAR technologies but it is well known that ambiental temperature and environment characteristics are directly influencing the response of these sensors.

On the other side, CSSs include encoders, electrical resistivity and strain sensors, capacitive flexible sensors, optoelectronics sensors, fiber optic sensors (FOSSs) and Micro/Nano Electro-Mechanical Systems (MEMS/NEMS) Inertial Measurement Unit (IMU) sensors. Encoders are well established solutions that provide the angular movement of joints but if compact designs are needed, their integration could be a major problem. Moreover, if the hyper-redundant robot has many DoF and encoders need to have a high number of pulses per turn, the cost of such a solution can increase drastically [18]. Electrical resistivity and strain sensors are more suited for gaming and wearables technology, require many cables, and their installation can be costly [19]. An alternative technology is that of flexible soft sensors. For example, Bend Labs [20] developed a set of flexible sensors based on differential capacitance measurement of angular displacement. They claim their pioneering technology is capable of sub-degree resolution and does not suffer from drift. Optoelectronic sensors can sense the shape in real-time and are a combination of light sensors, gyroscopes, and accelerometers [21]. For example, Dementyev et al. [22] developed a low-cost contact-based optoelectronic shape sensor called SensorTape suited for posture measurement. FOSSs is an emerging sensing technology that enables 3D shape reconstruction by using multi-core optical fiber cable together with strain sensors with a submillimeter accuracy [16,17]. As an example, Schmitz et al. presented in [23] an articulated single joint surgery snake-like robot prototype with applications in minimally invasive surgery. Their results indicate a mean absolute error (MAE) of 0.71 deg over a 35 deg bending range. Although FOSSs is a very promising shape sensing technology, the majority of works present proof-of concept solutions in medical/geotechnical applications, and it is well accepted in the literature that many technical issues must be overcome to become a mature technology. For a comprehensive review of FOSSs and its applications the reader should consult [16,17].

MEMS/NEMS IMU sensors have become very accessible in the last years due to the miniaturization and advances in MEMS/NEMS technologies and are increasingly used as shape sensing solutions in many robotic applications [24,25,26]. For example, one encoder can be replaced by an IMU sensor reducing the cost up to five times [18]. IMU sensors can be found in packages with 6-DoF (a 3-axis accelerometer and a 3-axis gyroscope) or 9-DoF (combined with an extra 3-axis magnetometer to work as an Attitude and Heading Reference System (AHRS) [27]). However, integration drift, magnetic disturbances, electro-magnetic noise, and calibration procedures are the main issues when it comes to IMUs, but such problems are usually solved by (1) fusing inertial measurements with additional information from kinematic mathematical models and (2) by using extensive signal processing and well-established digital filtering techniques such as Extended Kalman Filtering (EKF) or Unscented Kalman Filtering (UKF) [28,29,30,31,32]. Thus, stable data output can be measured with a precision as high as 0.05 deg for X-Y axes and 1 deg for Z axis as reported in [33]. In addition, their reliability was confirmed in studies such as barbell velocity assessments [34], pervasive healthcare applications [35], online kinematic calibration of robot manipulators [36] or wireless body area networks [37].

Concerning the use of IMUs in snake-like hyper-redundant robots, Kang et al. [38] validated a closed-form kinematic model of a continuum robot actuated pneumatically using an Xsens Mti-30 IMU sensor attached to the end-effector. The results showed that pitch and roll angle were estimated by the model with an error of 1 deg. In [39], Peng et al. developed a three-section continuum manipulator (lower, middle, and upper joint) actuated by nine pneumatic artificial muscles. To validate the kinematic and compliance models they used three 6-DOF IMUs attached at the end of each section to measure bending angle, acceleration, and rotation parameters. The errors obtained at the end-effector were less than 7 mm, one source of these errors was related with sensors measurements as highlighted by the authors. In [40], Zhang et al. developed a snake-like robot prototype with micro-inertial sensors. They attached a BSN-Body Sensor Network (a 3D accelerometer ADXL330 and a 3D gyroscope ITG-3200) on each segment of the robot and used a proprietary algorithm to estimate joint angles accurately. The readings from BSNs were compared with the reading from the on-bord encoders. A Root Mean Square Error (RMSE) error between 0.4–0.47 deg and between 1.17–1.22 deg was reported for yaw and pitch angle, respectively, for different elements of the robot. In [41], Luo et al. proposed a fusion pose estimation method (RBF-UKF) for a redundant robot that is based on a multisensory fusion approach that is applied in two phases: a “pre-enhancement” fusion phase where information from a RGB-D camera and a MARG (Magnetic, Angular Rate, and Gravity) sensor are fused with the information from an optical encoder and an adaptive fusion phase where the pose of the robot is predicted and various parameters are adaptively adjusted. Their experimental setup consists of eight modules with 1-DoF serially connected as a redundant manipulator. Experimental results showed that RMSE error for pose estimation with EKF and UKF filtering methods were four times higher than their proposed RBK-UKF method. In another paper, Zhao et al. [42] proposed an autonomous navigation method for a joint-based snake-like robot considering robot’s motion characteristics, a strapdown inertial navigation system, and sensor fusion techniques using EKF filtering. Various experiments were conducted (from linear motion to circular motion) and position error was less than 5% of total traveled distance of the robot.

As can be seen from the analysis, various sensing technologies can be used for hyper-redundant robots, but IMUs is highlighted as a promising solution in many research papers. Therefore, although IMUs lead to larger errors than those obtained with encoders, advantages such as miniaturization, low energy consumption, reduced costs, and small weight, make IMUs an appropriate candidate for certain applications of snake-like hyper-redundant robots where the problem of shape control is important for navigation in unstructured environments. If the end effector of the robot integrates an additional motion sensor that helps to maintain the robot’s end effector on the planned trajectory, a deviation from the planned shape can be accepted (with a certain margin of error).

In this context, the paper addresses the problem of shape sensing and pose estimation of an articulated joint-based snake-type robot (called Python), that is designed to operate in unstructured environments under various uncertainties. The original contributions are related to (1) the robots shape sensing computational system and (2) the Hard-ware-in-the-Loop (HIL) testing method.

The shape sensing computational system is a proprietary algorithm custom designed for a Python robotic system but can be used also for other hyper-redundant robots. It addresses the problem of kinematics and shape sensing of the robot (using a network of IMU AHRS), as a part of the control system useful in path planning and navigation tasks (in unstructured environments). The computational system integrates a dedicated network of AHRS IMU sensors and a shape sensing algorithm that runs in real time on a microcontroller-based board. The IMU sensor network is embedded into the structure of the robot and communicates using CAN network, thus resulting in a compact design with a reduced number of communication wires. The proposed sensing algorithm uses the data from the sensor network to directly calculate the kinematic parameters of the robot in the modules’ operational space reducing in this way the computational time.

The novel testing method is based on the HIL technology and allows real-time testing and assessment of algorithms used for calculus of robot shape and pose for hyper-redundant robots that integrate a sensory network. The method uses the information from the sensors of a real robot, but the estimation of shape and position are made using a virtual model/robot. The same information is entered into the robot’s position and shape computational algorithm, and the results are compared. Once validated, the algorithm could be used by the real robot as part of the robot’s control strategy.

The advantage of this method is that the shape algorithm validation could be performed without the need of an external sensory system to measures the pose of each robot platform/module. The flexibility of the Simscape platform also allows for the implementation of different robotic structures, thus the method could be applied to other similar robot topologies. Extending this setup, the method could also be used to test the entire control strategy of the robot, benefiting from the advantages of HIL simulations.

The paper is structured as follows. Chapter 2 presents the conceptual design of the Python robot that can be used for dexterous manipulations in unstructured environments, while in Chapter 3 details are given about the advanced real-time feedback system integrated in Python for shape sensing and pose estimation. A HIL Simulation scenario is presented in Chapter 4 where the experimental results from the experimental setup are compared with the simulation result of a virtual model of the robot that is running in parallel on a dSPACE HIL simulator. Finally, the paper ends with the conclusion.

## 2. Robot Structure Description and Kinematics

**Figure 1:**
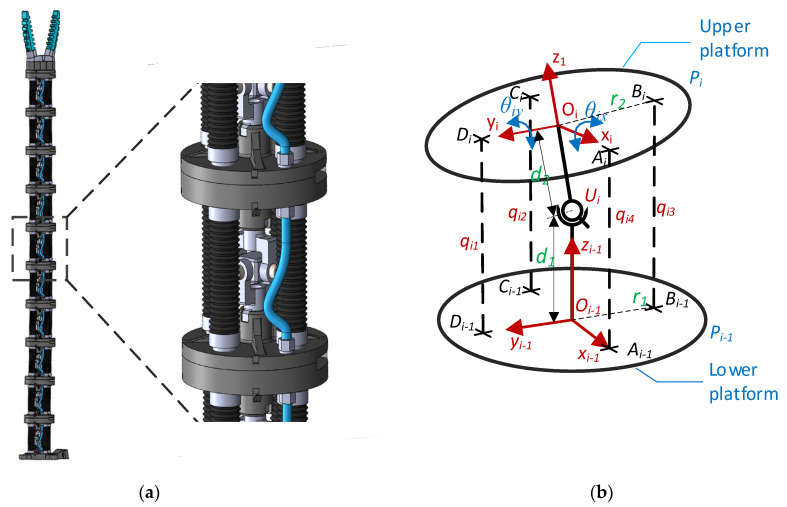
Python Robot (**a**) CAD Model with *n* = 10 modules (**b**) kinematic diagram of one module.

The Phyton robot has a hyper-redundant structure that consists of a set of *n* (*n* = 10 ÷ 20) identical modules that are serially connected (Figure 1a). On the last module a soft gripper and/or an inspection camera can be connected, allowing thus the implementation of tasks related to harvesting and inspection for a predefined range of crops cultivated in vertical farms.

Each module of the robot has two platforms that are connected through a universal joint *U_i_* {*i =* 1…*n*} that is at the distance *d*_1_ from the lower platform and *d*_2_ from the upper platform. The actuation of the structure is performed by four bellows actuators that are working in tandem (two by two). The actuators are connected on the lower and upper platforms on the positions indicated by the points *A_i_/A_i_*_−1_, *B_i_/B_i_*_−1_, *C_i_/C_i_*_−1_ and *D_i_/D_i_*_−1_ (Figure 1b). Thus, each module has 2-DoF (two rotations along the axis *O_ix_* and *O_iy_* {*i =* 1…*n*}), respectively, for *n* = 10 the whole structure will have 20-DoF. The end effector position and orientation are characterized by the following parameters: *Gx*, *Gy*, *Gz* and *G_α_*, *G_β_*, *G_γ_*.

**Figure 2:**
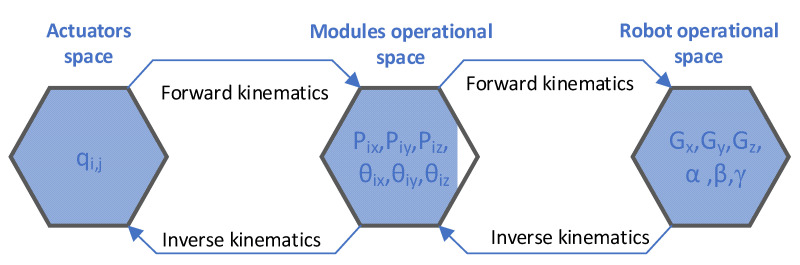
Kinematic analysis of hyper-redundant robots [43].

In order to control the robot behavior and implement the path planning strategies the kinematic parameters of the entire structure must be determined (robot shape sensing). For this type of robots, the kinematics can be expressed as a successive mapping between actuator space, module operational space and robot operational space [43,44] (Figure 2). In the paper a new approach is proposed, as presented in [45] by the authors, where the kinematic is implemented by mapping only between module operational space and robot operational space. This is achieved by fusing the data from the robot sensor network and determining the kinematic parameters of the upper platforms in each module directly for all the robot’s modules.

The proposed method uses the orientation data for the upper and lower platform, that are provided by the sensory system and the geometric characteristics of the modules to calculate the direct kinematics of the robot. The algorithm receives the Euler angles *α_i_*, *βi* and *γ_i_* {*i =* 0*…n*} that define the orientation of all the platforms *P_i_* {*i* = 0*…n*}. The rotation sequence that defines the orientation of each platform is *z-y-x*. The rotation matrix for each platform is determined by using (1).

(1) $$R_{Pi}=\left[\begin{array}{ccc} r_{11} & r_{12} & r_{13}\\ r_{21} & r_{22} & r_{23}\\ r_{31} & r_{32} & r_{33} \end{array}\right],\;\left(i=0\ldots n\right)$$

For each *R_Pi_* {*i* = 0…*n*} matrix, the *r_kl_ {k =* 1…3; *l =* 1…3} components are calculated based on the *α_i_*, *βi* and *γ_i_* {*i* = 0…*n*} Euler angles, using the following equations:

(2) $$r_{11}=\cos\left(\gamma_{i}\right)\cos\left(\beta_{i}\right)\;r_{12}=\cos\left(\gamma_{i}\right)\sin\left(\beta_{i}\right)\sin\left(\alpha_{i}\right)-\sin\left(\gamma_{i}\right)\cos\left(\alpha_{i}\right)\;\;r_{13}=\cos\left(\gamma_{i}\right)\sin\left(\beta_{i}\right)\cos\left(\alpha_{i}\right)+\sin\left(\gamma_{i}\right)\sin\left(\alpha_{i}\right)\;r_{21}=\sin\left(\gamma_{i}\right)\cos\left(\beta_{i}\right)\;r_{22}=\sin\left(\gamma_{i}\right)\sin\left(\beta_{i}\right)\sin\left(\alpha_{i}\right)+\cos\left(\gamma_{i}\right)\cos\left(\alpha_{i}\right)\;r_{23}=\sin\left(\gamma_{i}\right)\sin\left(\beta_{i}\right)\cos\left(\alpha_{i}\right)-\cos\left(\gamma_{i}\right)\sin\left(\alpha_{i}\right)\;r_{31}=-\sin\left(\beta_{i}\right)\;r_{32}=\cos\left(\beta_{i}\right)\sin\left(\alpha_{i}\right)\;r_{32}=\cos\left(\beta_{i}\right)\cos\left(\alpha_{i}\right)$$

The *R_Pi_ {i =* 0…*n}* rotation matrices are used to calculate *R_Rj_ {j =* 1…*n}* rotation matrices that defines the relative rotation between two consecutive platforms (*P_j_* and *P_j_*_−1_).

(3) $$R_{Rj}=R_{Pj}*transpose\left(R_{Pj-1}\right)\;;\{j=1\ldots n\}\;$$

The *R_Rj_ {j =* 1…*n}* rotation matrices are then used to extract the relative rotation angles *θ_jx_* and *θ_jy_* between two consecutive platforms *P_j_* and *P _j_*_−1_ {*j =* 1…*n*} (Figure 1b). Due to the universal joint between the two platforms, the relative rotation *θ_jz_* along the axis *O_jz_* is always zero. The *θ_jx_* and *θ_jy_* angles are calculated using the following equations:

(4) $$\begin{array}{c} \theta_{jy}=atan2\left(-R_{Rj}\left(3,1\right),\sqrt{R_{Rj}{\left(1,1\right)}^{2}+R_{Rj}{\left(2,1\right)}^{2}}\;\right)\\ \theta_{jx}=atan2\left(\frac{R_{Rj}\left(3,2\right)}{\cos\left(\theta_{yi}\right)},\frac{R_{Rj}\left(3,3\right)}{\cos\left(\theta_{yi}\right)}\right) \end{array}$$

where: *R_Rj_* (1,1), *R_Rj_* (2,1), *R_Rj_* (3,1), *R_Rj_* (3,2) and *R_Rj_* (3,3) are the elements of *R_Rj_* rotation matrix.

Using the robot geometric dimensions and the relative angles between two consecutive robot platforms, the position and orientation of all mobile platforms *P_j_* {*j* = 1…*n*} can be calculated with respect to the world frame. For this purpose, an iterative process, starting from the base (platform *P_0_*) is implemented using a set of homogenous transformations. The following general equation is used:

(5) T0j=T0j−1Tj−1j {=1…n}

At each module level (two consecutive platforms), the homogenous transformation matrix Tj−1j includes one translation (along *O_jz_* axis with *d*_1_), two orientations (along *O_jx_* and *O_jy_* axis with *θ_jx_* and *θ_jy_* angles) and one translation (along *O_jz_* axis with *d_2_*). Equation (6) describes this process:

(6) Tj−1j=[100010001Pd101][Rθjx,θjy 00001][100010001Pd201]

Based on the homogenous transformations T0j for *P_j_* {*j =* 1…*n*} platform, the position and orientation relative to the global coordinate system can be expressed using (7) and (8).

(7) Pjx=T0j(1,4)Pjy=T0j(2,4)Pjx=T0j(3,4)

(8) Pjα=atan2(T0j(3,2)cos(Pjβ),T0j(3,3)cos(Pjβ))Pjβ=atan2(−T0j(3,1), (T0j(1,1))2+(T0j(2,1))2 Pjγ=atan2(T0j(2,1)cos(Pjβ),T0j(1,1)cos(Pjβ)))

The end effector pose is calculated using the T0nG (9) homogenous transformation matrix in the same way as presented above. The pose of the end effector characteristic point *G* (*Gx*, *Gy*, *Gz* and *G_α_*, *G_β_*, *G_γ_*) relative to *P_n_* platform, is determined by adding additional translation and/or rotations $T_{G}$ depending on the geometric characteristics of the gripper/camera that is connected to the robot.

(9) T0nG=T0n TG

The presented algorithm is next implemented in MATLAB/Simulink and the generated code runs on Discovery microcontroller-based board as part of the proposed shape sensing computational system. The algorithm runs in real time and the results are displayed on a dedicated Graphical User Interface (GUI).

## 3. Design and Implementation of the Shape Sensing Computational System

**Figure 3:**
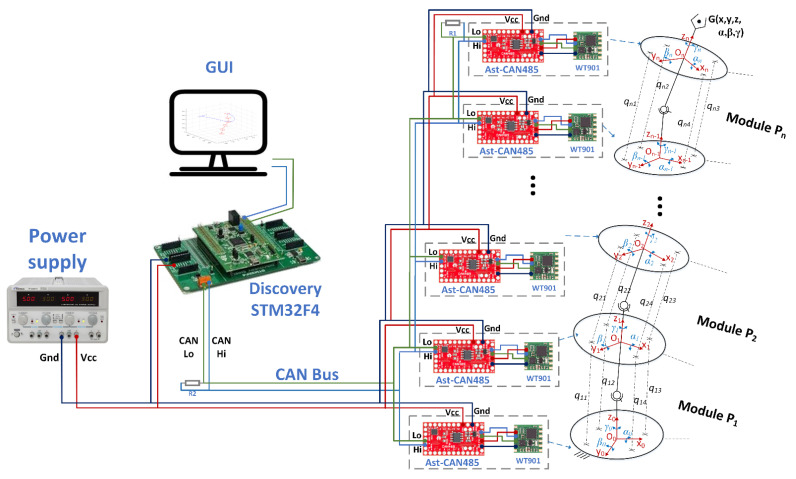
Schematic of the shape sensing computational system.

The main components of the shape sensing computational system are presented in Figure 3. The system integrates: the dedicated network of AHRS IMU sensors; the shape sensing algorithm that runs in real time on the Discovery board and the robot GUI.

The sensor network includes the nine-axis attitude angle sensors (model WT901 produced by WitMotion [33]) that are attached to each robot module. Each AHRS IMU sensor is connected to an AstCAN 485 microcontroller board and the communication between the two components is implemented using the UART interface. All the AstCAN boards are interconnected using a CAN network, allowing exchange in real time of information between the central control system (Discovery board) and each module. The sensors provide the orientation of each platform (the attitude angles: *α_i_*, *βi* and *γ_i_* {*i =* 0…*n*}) using the Northeast sky coordinate system [33]. The WT901 sensors are used in the proposed shape sensing computational system due to their high precision in measuring the angles along *X* and *Y* axis of 0.05 deg. The precision along *Z* axis is 1 deg and the maximum data output frequency from each sensor is 100 Hz. For calculating the attitude, the IMU sensor integrates a high-precision gyroscope, an accelerometer and a geomagnetic sensor. The accelerometer range was set to 16 g and the gyroscope maximum speed was set to 2000 deg/s. The sensors data are locally fused using a dynamic Kalman filter algorithm with a bandwidth of 20 Hz.

The sensor data, provided by each robot module, are further processed by the Discovery board that runs the proposed shape-sensing algorithm. The algorithm uses the sensors data to detect the shape and pose of the end effector. The software was developed using MATLAB/Simulink, and the code was generated automatically using Simulink Coder. The execution time of the code is less than 50 ms and the main program sampling period is 20 Hz. The obtained numerical results are displayed on the robot GUI that runs on a PC. The GUI was developed using MATLAB Guide tool and communicates with the Discovery board using the UART interface.

**Figure 4:**
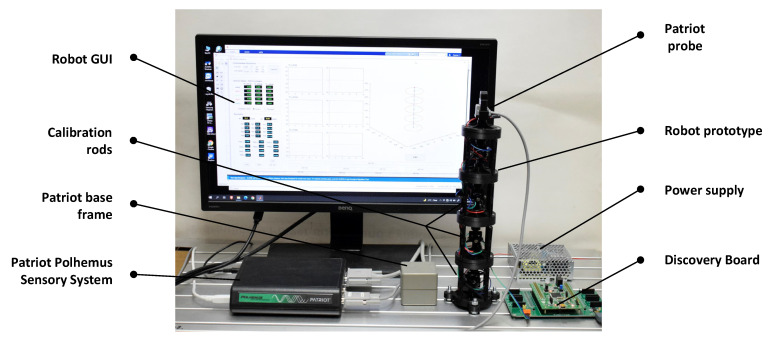
Experimental Stand—Robot prototype with *n* = 4 modules.

For testing the proposed shape sensing computational system, an experimental stand was developed as can be seen in Figure 4. The stand includes: the prototype of the proposed hyper-redundant robot with a total of 8-DoF (number of modules *n* = 4), the shape sensing computation system previously described and the Patriot Polhemus Sensory System (used for testing purposes). As mentioned before, for each module a WT901 attitude sensor is attached to the lower and upper platform resulting in a total of 5 AHRS IMU sensors embedded in the structure of robot prototype, which are used to determine the shape of the robot.

The calibration process of the system takes into account the calibration of each IMU sensor used in the sensory network and the calibration of the robot structure after mounting all the sensors. The IMU calibration is performed before mounting on the robot and consists in accelerometer calibration and magnetic calibration based on the methodology described in the WT901 sensor manual [33]. The robot calibration aims at eliminating any mounting errors of the sensors. Therefore, a set of rods were designed and mounted on the robot structure during the calibration process. The rods position each robot platform parallel to the base frame (as presented in Figure 4). In this position the roll and pitch should be zero and the differences recorded from the sensor data are stored and added as bias by the Discovery board. The alignment along z axis is also analyzed and calibrated.

## 4. Experimental Results

### 4.1. Testing Methods

The effectiveness and performance of the robot shape sensing computational system is evaluated using two testing methods. The first method is based on HIL technology and uses a virtual model to validate the shape sensing algorithm (robot shape, end-effector pose). The second method uses an external sensory system to validate the output of the shape sensing computational system in terms of end-effector pose.

The first testing method uses a HIL technology to evaluate the shape sensing algorithm. HIL (Hardware-in-the-loop) simulations are defined as synergetic combinations between physical and virtual components which allow development of experiments where real and virtual components run in the same application [46]. The evaluation of the efficiency (precision) of the shape sensing algorithm is important because it is the basis of the control strategy (as part of the control system). In the proposed method the data provided by the robot sensors are used to detect the shape of the robot by the shape sensing algorithm which runs on a Discovery board. The same data are transmitted to a virtual model (that runs on dSPACE) which is used to validate the effectiveness of the algorithm. If large errors occur between these two approaches, they can only be caused by the shape sensing algorithm because the virtual model is built based on a certified modeling technology (MATLAB/Simscape) and simulated on a real-time platform (dSPACE). Small errors may occur due to different discretization or different calculation precision specific to simulation platforms (Discovery vs. dSPACE).

**Figure 5:**
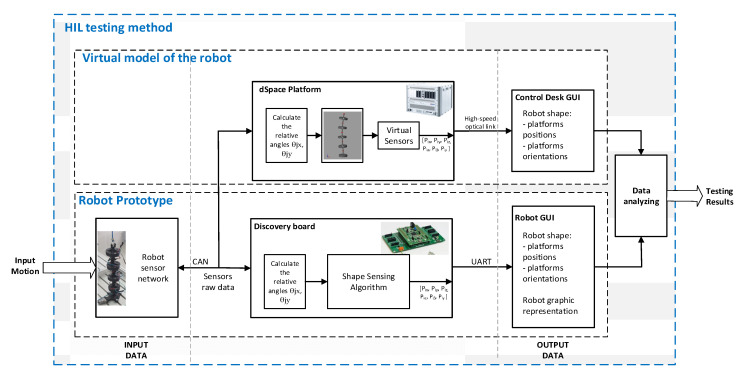
Conceptual diagram for the proposed testing method.

A conceptual diagram for the proposed testing method setup is presented in Figure 5. In this experimental setup the robot prototype is running in parallel with a virtual robot model that is implemented on dSPACE DS1006 HIL Platform and the numerical results obtained are analyzed and compared. In order to evaluate the precision of the shape sensing computational system, the MAE error is calculated.

The data processing flow in the proposed method is as follows. The raw data (Input Data) provided by sensors are used as inputs in the virtual model which return the position and orientation of each platform through virtual sensors and implicit kinematics of the virtual robot model. The virtual model of the robot is implemented using Simulink/Simscape blocks and replicates the prototype of the real robot. As part of the model, dedicated virtual sensor blocks for each platform that form the modules of the robot are implemented. Thus, the correct readings on the platform’s position and orientation are obtained. The results (Output Data) are displayed using ControlDesk (dSPACE GUI interface). The Output Data calculated by using the proposed shape sensing algorithm that runs on Discovery board are analyzed and compared with the ones obtained from the virtual robot that runs on the dSPACE platform. The obtained data are saved using the GUI interfaces and the results are analyzed in MATLAB.

**Figure 6:**
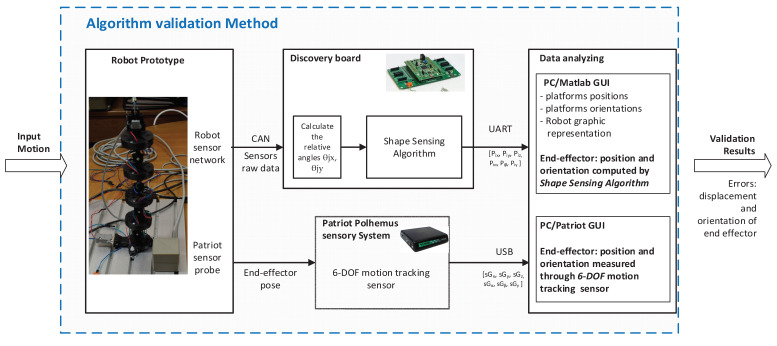
Conceptual diagram of the testing method for the end-effector pose.

The second testing method is used to validate the shape sensing computational system by measuring the end-effector pose. The conceptual diagram of the method is presented in Figure 6. In this setup the Patriot Polhemus sensory system is used to measure the end effector position and orientation. For measuring these parameters, the Patriot sensory system uses one base frame (electromagnetic field source) that is attached to the fixed frame of the robot and a probe that is mounted on the end effector on the robot. The sensors’ base frame produces its own electromagnetic field that is used to calculate the probe’s relative distance and orientation. The sensor provides data with a resolution of 0.08 mm and 0.016 deg at a 60 Hz rate for measurement setups where the distance between the probe and fixed base is less than 609 mm (in this setup the average distance was 350 mm between the two elements) [47]. The data provided by the Patriot sensory system (parameters: *sGx*, *sG_y_*, *G_z_*, *sG_α_*, *sG_β_*, *sG_γ_*) are displayed and stored using the Patriot GUI interface. These data are then compared with the values calculated by using the proposed sensing computational system (parameters: *G_x_*, *G_y_*, *G_z_*, *G_α_*, *sG_β_*, *sG_γ_*) which are saved using the robot GUI interface.

### 4.2. Validation of Experimental Results

**Figure 7:**
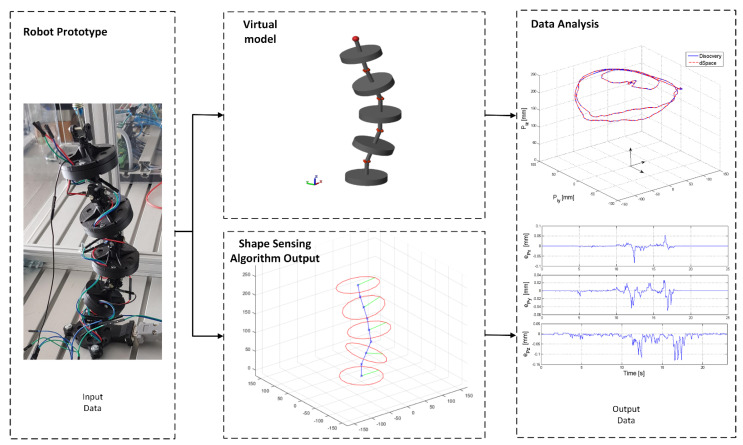
HIL testing method—validation of experimental results.

Using the experimental setup presented in Figure 5, a set of experiments were developed in order to measure and compare: relative angles between platforms, position and orientation of the platforms and trajectory of the end effector. In the experiments, an arbitrary continuous movement was manually imposed to the robot modules. The variations of the movements’ amplitudes and platform orientations aimed at covering multiple regions of the robot workspace, thus assuring a set of representative data. The sensors’ data were sent to dSPACE Platform and Discovery board as input data for calculating the robot shape (Figure 7).

**Figure 8:**
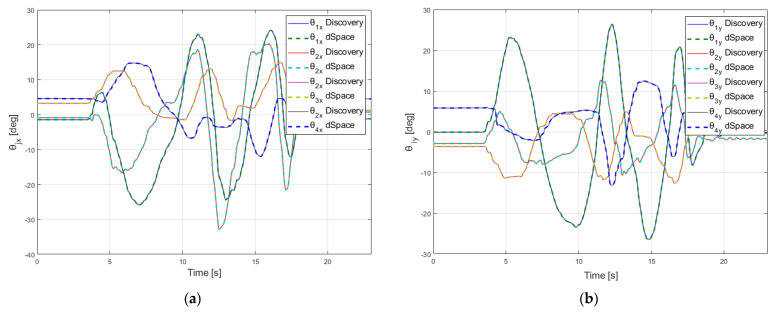
Variation of the relative angles between platforms (**a**) angle *θjx* (**b**) angle *θjy*.

**Figure 9:**
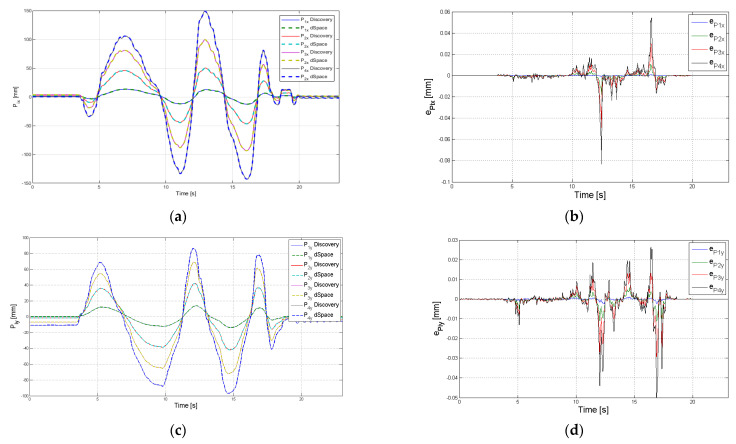

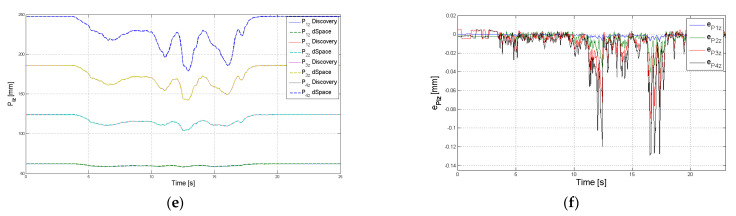
Experimental results for the position of each module of the robot: (**a**) parameters *P_jx_* (**b**) absolute error of *P_jx_* (**c**) parameters *P_jy_* (**d**) absolute error of *P_jy_* (**e**) parameters *P_jz_* (**f**) absolute error of *P*_jz_.

**Figure 10:**
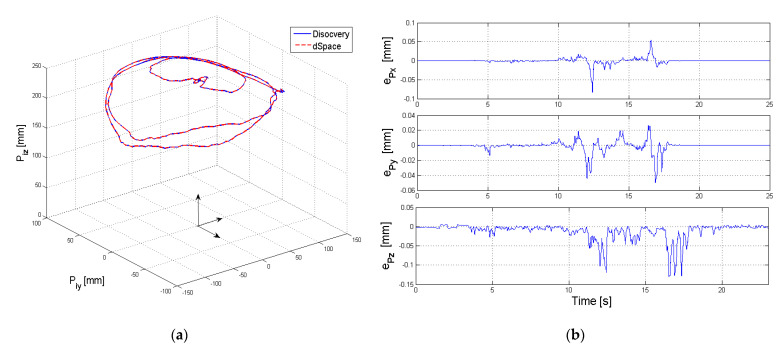
Trajectory of the end effector (4th module) (**a**) 3D representation (**b**) absolute positioning error along axis *O_x_*, *O_y_* and *O_z_* for the 4th module.

**Figure 11:**
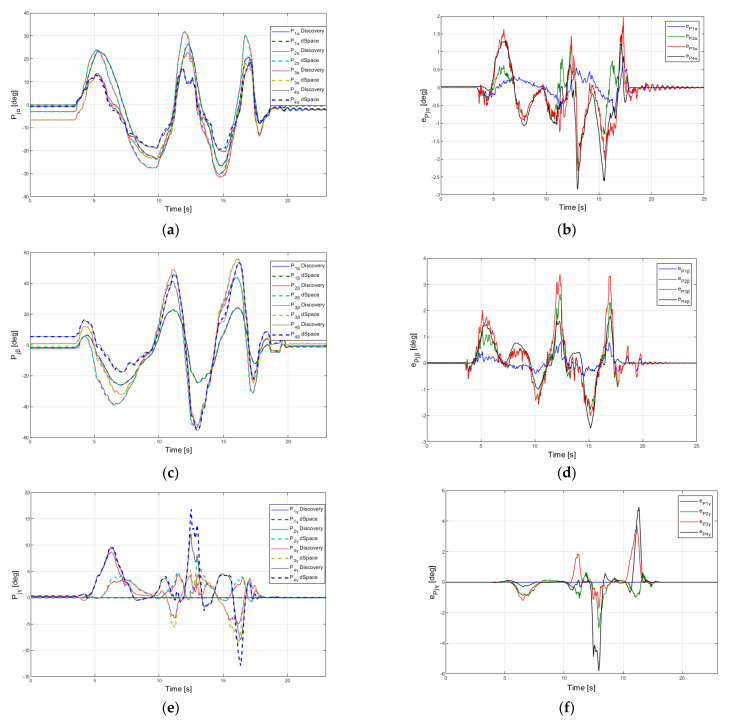
Experimental results for the orientation of each module of the robot for (**a**) parameter *P_jα_* (**b**) absolute error of *P_jα_* (**c**) parameter *P_jβ_* (**d**) absolute error of *P_jβ_* (**e**) parameter *P_jγ_* (**f**) absolute error of *P*_jγ_.

The experimental results are presented in Figure 8, Figure 9, Figure 10 and Figure 11. In these figures, the values calculated using Discovery board are represented with continuous line and the values measured using dSPACE platform are represented with dash lines.

The raw data that are transmitted through the CAN sensor network, are used to calculate the relative angles between all the robot’s consecutive platforms. In Figure 8, the variations of the angles *θ_jx_* and *θ_jy_* {*j* = 0…4} are presented for each module. The dSPACE and Discovery boards calculate these angles independently and the maximum of the absolute errors between these signals is negligible (less than 10^−6^ deg) being affected only by the sample time used by each of the two environments.

The variation of the position of each platform is presented in Figure 9 (parameter *P_jx_* {*j* = 1…4} in Figure 9a, parameter *P_jy_* {*j* = 1…4} in Figure 9c and parameter *P_jz_* {*j* = 1…4} in Figure 9e. The absolute error for each platform for the three parameters is presented in Figure 9b,d,f. The obtained maximum absolute error for *P_jx_* parameter is 0.083 mm, for *P_jy_* is 0.049 mm and for *P_jz_* parameter is 0.129 mm. As can be seen, the absolute errors are higher in the upper platforms, which is caused by a much bigger amplitude of position variation for these modules in comparison with the modules at the robot base.

The position parameters *P_jx_*, *P_jy_*, *P_jz_* {*j* = 1…4} are used to reconstruct the robot shape. A 3D representation of the structure is represented in real time in the robot GUI interface that displays the data calculated by the Discovery board (Figure 4). The MAE errors for the fourth module were calculated, the obtained values are: for *P_4x_* the MAE error is 0.0028 mm at a variation of 292.4 mm, for *P_4y_* the MAE error is 0.003 mm at a variation of 183.5 mm and for *P_4z_* the MAE error is 0.0116 mm at a variation of 68.9 mm.

The 3D representation of the end effector trajectory (position of the characteristic point for the 4th module {*P_4x_*, *P_4y_*, *P_4z_*}) is presented in Figure 10a.

The variations of the platform’s orientations are presented in Figure 11 (parameter *P_jα_* {*j* = 1…4} in Figure 11a, parameter *P_jβ_* {*j* = 1…4} in Figure 11 c and parameter *P_jγ_* {*j* = 1…4} in Figure 11d. The MAE errors for parameter *P_jα_* (Figure 11b,d,e) are 0.404 deg at a variation of 40.4 deg, for parameter *P_jβ_* is 0.365 deg for a variation of 109.1 deg and for parameter *P_jγ_* is 0.291 deg for a variation of 20.1 deg.

After the analysis of the results, for this set of experimental data, it was observed that MAE errors for position varied between 0.0028 and 0.0116 mm and for orientation between 0.291 and 0.404 deg for the fourth module of the robot. These values were directly affected by the amplitude of the movements of these parameters and the hardware characteristics of the two computational systems (different discretization, different calculation precision etc.).

**Figure 12:**
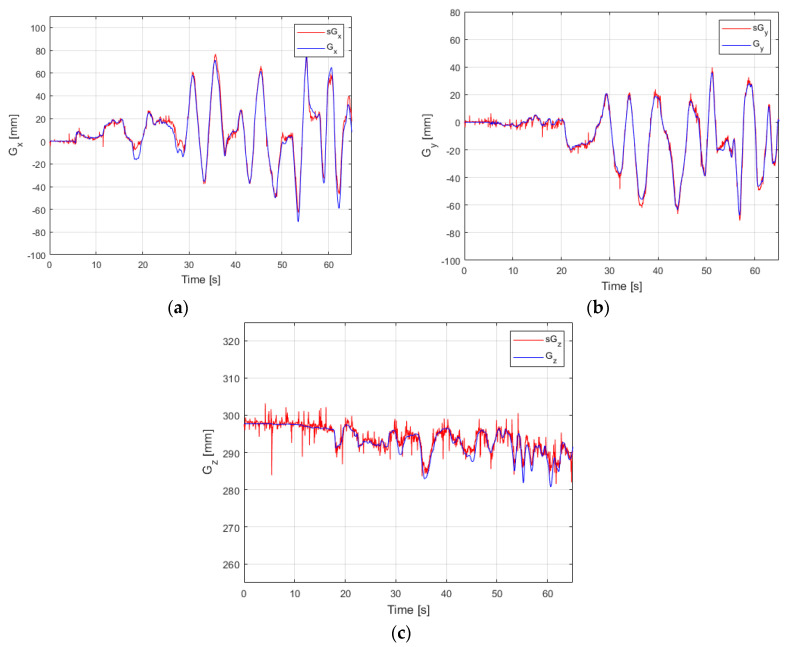
Experimental result—position of the end effector (**a**) parameter *G_x_* (**b**) parameter *G_y_* (**c**) parameter *G_z_*.

**Figure 13:**
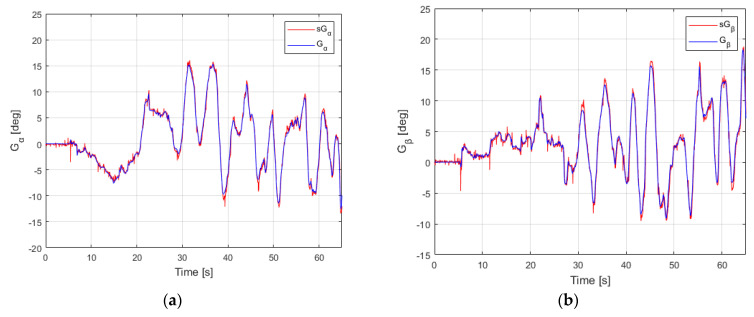

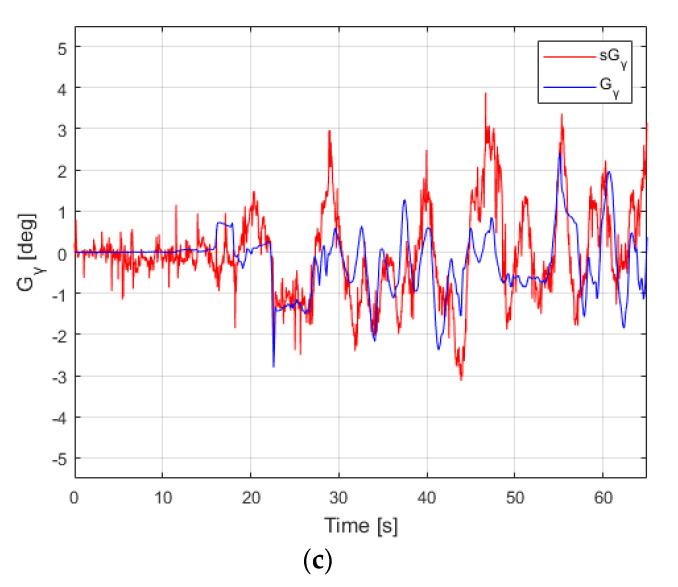
Experimental results—orientation of the end effector (**a**) parameter *G_α_* (**b**) parameter *G_β_* (**c**) parameter *G_γ_*.

By using the second testing method (Figure 6), a set of experiments was developed to validate shape sensing computational system by measuring the end-effector pose. The experimental data results used in the validation process are presented in Figure 12 and Figure 13. In these figures, the data measured by using the Patriot sensor are represented with a red line and the data calculated by using the shape sensing computational system proposed in the article with a blue line.

The MAE error obtained for parameter *G_x_* is 2.91 mm at a variation of 147.4 mm, for parameter *G_y_* is 1.52 at a variation of 103.3 mm and for parameter *G_z_* the error is 0.539 mm for a range of 17.4 mm.

The MAE error obtained for parameter *G_α_* is 0.33 deg at a range of 27.8 deg, for parameter *G_β_* is 0.36 at a range of 27.4 deg and for parameter *G_γ_* is 0.36 at a range of 5.2 deg.

During the experiments, it was observed that the orientation sensing along *O_z_* axis for this type of sensors was influenced by the presence of electromagnetic waves. In this context, design restrictions are imposed concerning the electronic/magnetic components that are not allowed to be present in the closed proximity of the sensor.

An advantage of using this type of sensors is that the cumulative errors are reduced, due to the fact that the orientation between the platforms of each module is calculated independently based on the sensor data that are absolute values.

Analyzing the numerical results obtained with the two testing methods it was observed the follows. Using the HIL testing method, the MAE errors obtained for positioning were less than 0.0116 mm and for orientation were less than 0.404 deg when the robot shape was evaluated. Related to evaluation of the end effector pose, using the second testing method, it was observed that the MAE errors were less than 2.91 mm for X, Y, Z position and less than 0.36 deg for orientation angles. Taking into account the obtained results (effectiveness and performance) it can be concluded that the shape sensing computational system can be used as part of the control system of the Python robot.

## 5. Conclusions

The paper presented a new approach for shape sensing of a hyper redundant robot with articulated joint-based rigid structure. A shape sensing computational system (which integrates a dedicated network of AHRS IMU sensors and a shape sensing algorithm) was proposed and developed and two testing methods were implemented for validating its effectiveness and performance.

The proposed computational system offers several advantages: reduced computational time, which makes the algorithm more feasible for real time computation; reduced cumulative errors due to the absolute measurements of the sensor network, improved robot design related to the system dimensions and communication buses.

The proposed HIL testing method uses a virtual robot which runs on a real time HIL simulation platform (dSPACE), in parallel with the computational system. The experimental data outputs were compared and the results validate the proposed algorithm. In the experiments developed, the MAE errors for positioning were less than 0.0116 mm and for orientation were less than 0.404 deg. The results obtained offer a good perspective for using the proposed sensor network in the development of the robot with application in harvesting and inspection tasks.

The second testing method was used to capture the pose of the end-effector with a state-of-the-art motion capture system and then it was compared with the one provided by the shape sensing computational s47ystem proposed in this paper. Experimental results were analyzed and MAE errors were less than 2.91 mm for X, Y, Z position and less than 0.36 deg for orientation angles of the end-effector.

Both testing methods validated the proposed shape sensing computational system, which is further used to implement the Python robot control system. Other solutions to improve the shape sensing computational system performance could be addressed also. The authors intend to use flexible soft sensors from Bend Labs and to fuse the data received from the two sensor systems. This would allow an increase in precision and potential new application in domains where high accuracy is needed.

## Data Availability

Not applicable.
